# Evaluation of the cardiac subvolume dose and myocardial perfusion in left breast cancer patients with postoperative radiotherapy: a prospective study

**DOI:** 10.1038/s41598-023-37546-7

**Published:** 2023-06-29

**Authors:** Shan-Ying Wang, Kuan-Heng Lin, Yen-Wen Wu, Chih-Wei Yu, Shu-Ya Yang, Pei-Wei Shueng, Chen-Xiong Hsu, Tung-Hsin Wu

**Affiliations:** 1 Department of Biomedical Imaging and Radiological Sciences, National Yang Ming Chiao Tung University, Taipei, Taiwan; 2 Department of Nuclear Medicine, Far Eastern Memorial Hospital, New Taipei City, Taiwan; 3 Department of Radiation Oncology, Far Eastern Memorial Hospital, New Taipei City, Taiwan; 4 School of Medicine, National Yang Ming Chiao Tung University, Taipei, Taiwan; 5 Division of Cardiology, Cardiovascular Medical Center, Far Eastern Memorial Hospital, New Taipei City, Taiwan; 6 Department of Radiation Oncology, China Medical University Hsinchu Hospital, Hsinchu County, Taiwan

**Keywords:** Medical imaging, Breast cancer, Radiotherapy

## Abstract

Adjuvant breast radiotherapy could reduce the risk of local recurrence. However, the radiation dose received by the heart also increases the risk of cardiotoxicity and causes consequential heart diseases. This prospective study aimed to evaluate more precisely cardiac subvolume doses and corresponding myocardial perfusion defects according to the American Heart Association (AHA)’s 20-segment model for single photon emission computed tomography (SPECT) myocardial perfusion imaging (MPI) interpretation for breast cancer after radiotherapy. The 61 female patients who underwent adjuvant radiotherapy following breast cancer surgery for left breast cancer were enrolled. SPECT MPI were performed before radiotherapy for baseline study, and 12 months after for follow-up. Enrolled patients were divided into two groups, new perfusion defect (NPD) and non new perfusion defect found (non-NPD) according to myocardial perfusion scale score. CT simulation data, radiation treatment planning, and SPECT MPI images were fused and registered. The left ventricle was divided into four rings, three territories, and 20 segments according to the AHA’s 20-segment model of the LV. The doses between NPD and non-NPD groups were compared by the Mann–Whitney test. The patients were divided into two groups: NPD group (n = 28) and non-NPD group (n = 33). The mean heart dose was 3.14 Gy in the NPD group and 3.08 Gy in the non-NPD group. Mean LV doses were 4.84 Gy and 4.71 Gy, respectively. The radiation dose of the NPD group was higher than the non-NPD group in the 20 segments of LV. There was significant difference in segment 3 (*p* = 0.03). The study indicated that the radiation doses to 20 segments of LV in NPD were higher than those in non-NPD significantly at segment 3, and higher in other segments in general. In the bull’s eye plot combining radiation dose and NPD area, we found that the new cardiac perfusion decline may exist even in the low radiation dose region.

Trial registration: FEMH-IRB-101085-F. Registered 01/01/2013, https://clinicaltrials.gov/ct2/show/NCT01758419?cond=NCT01758419&draw=2&rank=1.

## Introduction

Current multimodality treatment strategies have markedly improved overall survival among patients with breast cancer, making long-term treatment-related changes in cardiac function a crucial concern for breast cancer survivors. Excessive radiation doses to the heart in breast cancer radiotherapy may result in life-threatening radiation-induced cardiac toxicity^1,2^.

With a detection sensitivity of up to 92%, single-photon emission computed tomography (SPECT) myocardial perfusion imaging (MPI) can detect myocardial defects at early stages and be used to predict the incidence of heart disease through semiquantitative cardiac function indices^3,4^. Hybrid imaging combining nuclear medicine functional information with anatomical computed tomography (CT) has been widely used nowadays. Furthermore, MPI with CT attenuation correction (AC) improved the issue of soft tissue attenuation assessment, especially at inferior wall and inferolateral wall myocardial perfusion defects^5–7^. New cadmium-zinc-telluride (CZT)-based SPECT cardiac scanner has higher energy resolution, which made efficient scans and EKG-gated data more applicable in daily practice^5,6^. In order to assess the correlation between cardiac radiation dose and early cardiac function decline, combining the CT-based radiation treatment plan with MPI could be crucial for monitoring breast cancer radiation treatment. Not only for the risk of chronic heart disease, such as coronary artery disease (CAD), microvascular disease or heart failure, moreover, to assess involved area more precisely^5,8^.

Darby et al.^1^ reported that mean heart dose (MHD) is linearly related to the risk of developing cardiovascular disease after radiotherapy, which increases by 7.4% with each additional 1 Gy of MHD. Roychoudhuri et al.^9^ discovered that the risk of cardiovascular disease following irradiation of the left breast was greater than that following irradiation of the right breast. In addition, cardiovascular mortality after radiotherapy for left-breast cancer is related to the radiation dose and volume received by the heart^10,11^.

Several studies have identified MHD as the preferable radiotherapy dose index for cardiac outcomes^12,13^. However, heterogeneity among MHDs and levels of radiosensitivity of the myocardium may affect the assessment of cardiotoxicity^14,15^. An increasing number of studies are investigating the effects of subvolume doses of radiation to the myocardium. Van den Bogaard et al.^16^ argued that the left anterior descending artery (LAD) dose and volume of the left ventricle (LV) that received ≥ 5 Gy (V_5_) should be included in the benchmarks for evaluating heart dose limits. Tang et al.^17^ divided the LV into 17 segments to investigate the differences among segmental doses according to the 17-segment model of the American Heart Association (AHA).

MHD cannot directly represent the dose received by each segment of the heart, nor can it be used as the only evaluation standard for heart dose limits. Therefore, this prospective study evaluated cardiac subvolume doses and corresponding myocardial perfusion defects according to the AHA’s 20-segment model for SPECT MPI interpretation for breast cancer after radiotherapy to delineate the correlation between precise dose distribution and cardiac perfusion assessment.

## Materials and methods

### Patients

**Figure 1:**
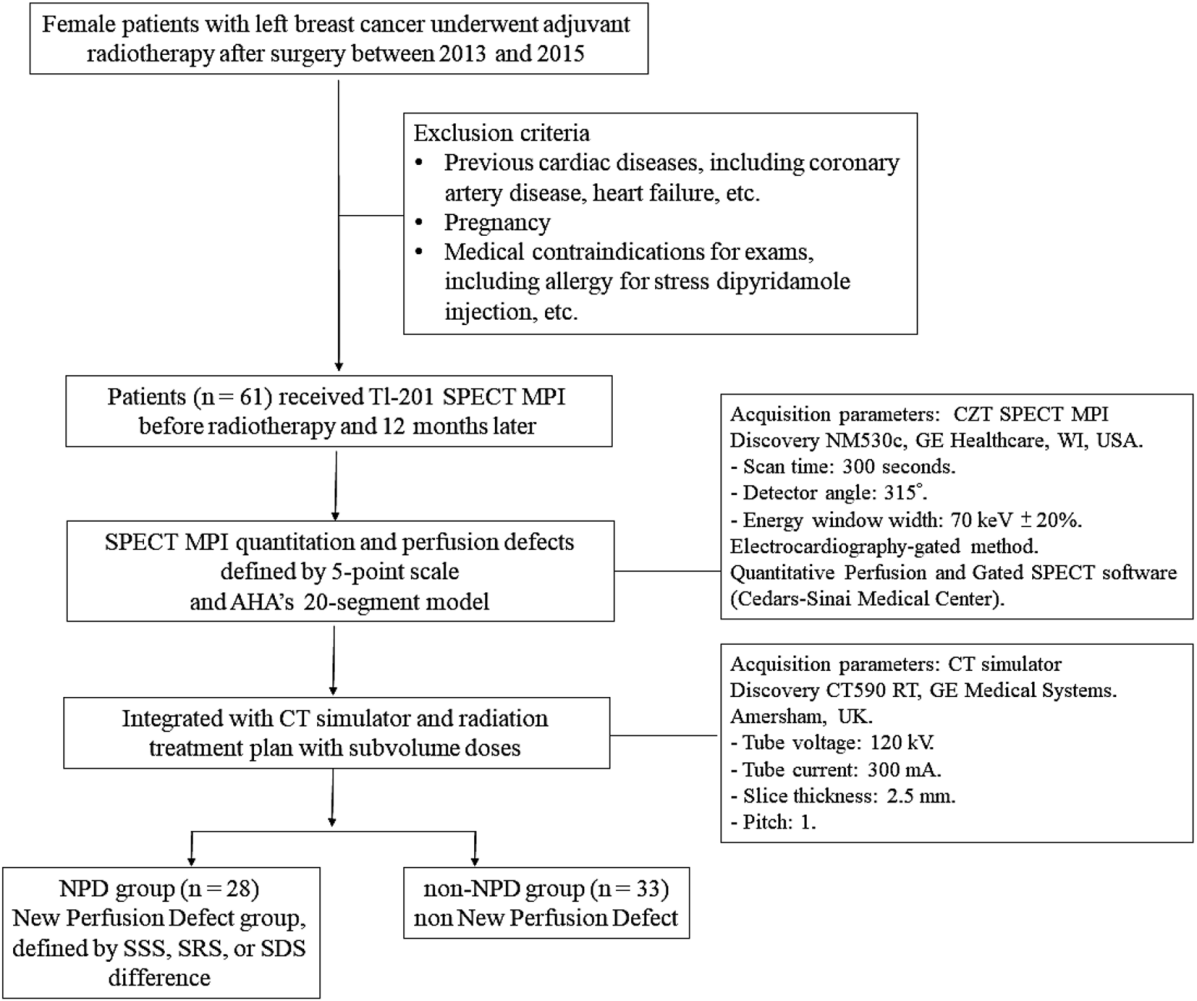
The flow diagram of the study. SPECT: single photon emission computed tomography. *CT* computed tomography. *MPI* myocardial perfusion imaging. *AHA* American heart association. *SSS* summed stress scores. *SRS* summed rest scores. *SDS* summed difference scores. *LV* left ventricle.

Between January 2013 and November 2015, we enrolled 61 female patients who underwent adjuvant radiotherapy following surgery for left-breast cancer. The patients underwent SPECT MPI before beginning radiotherapy and 12 months later for follow-up. The exclusion criteria were previous cardiac diseases, such as CAD and heart failure, pregnancy, and medical contraindications for the exam. This prospective study was approved by the Research Ethics Review Committee of Far Eastern Memorial Hospital (FEMH-IRB-101085-F). We confirm that all research was performed in accordance with relevant guidelines and regulations. All patients signed a written informed consent form before enrolment in the study. The flow diagram of patients’ enrollment is shown in Fig. 1. The baseline characteristics of the studied population are presented in Table 1, including basic demographic data, risk factors of CAD, and treatments received.

**Table 1:** Characteristics of patients.

Characteristic	All patients (n = 61)	NPD (n = 28)	non-NPD (n = 33)	*p* value
Age (years)				0.165
Median	50	52	50	
Range	29–65	29–65	32–54	
Risks of CAD				
BMI≧30 kg/m^2^	7 (11%)	2 (7%)	5 (15%)	0.336
Smoking	6 (10%)	3 (11%)	3 (9%)	0.835
Hypertension	9 (15%)	3 (11%)	6 (18%)	0.420
Hyperlipidemia	21 (34%)	7 (25%)	14 (42%)	0.158
Diabetes mellitus	6 (10%)	3 (11%)	3 (9%)	0.835
Treatment				
Surgery				0.409
BCS	39 (64%)	20 (71%)	19 (58%)	
MRM	22 (36%)	8 (29%)	14 (42%)	
Radiotherapy dose (Gy)				0.421
Median	55.00	55.00	55.00	
Range	40.05—61.00	50.00—61.00	40.05—60.00	
Chemotherapy	37 (61%)	18 (64%)	19 (58%)	0.600
Herceptin therapy	8 (13%)	2 (7%)	6 (18%)	0.209
Hormonal therapy	39 (64%)	14 (50%)	25 (76%)	0.069

*NPD* new perfusion defect, *non-NPD* non new perfusion defect, *CAD* coronary artery disease, *BCS* breast-conserving surgery, *MRM* modified radical mastectomy.

### Pharmacological stress SPECT MPI

All the patients underwent MPI with the standard pharmacological stress and SPECT scanning protocols^18^, by CZT SPECT gamma camera (Discovery NM530c, GE Healthcare, WI, USA). The patients were asked to avoid food and beverages containing caffeine or theophylline before the exam. Persantine (dipyridamole) was administered to the patients through intravenous injection according to the standardized stress protocol (0.56 mg/kg). A myocardial perfusion agent Tl-201 was administered at 7 min. The injection dose was 2, 2.5, or 3 mCi, according to each patient’s body weight (≤ 90, 90–100, or > 100 kg, respectively). Each patient’s post-stress and redistribution images were acquired and then analyzed. The scan time was 300 s; the detector angle was set at 315°; the energy window width was set to 70 keV ± 20%. Electrocardiography-gated method was applied.

### SPECT MPI quantitation and myocardial perfusion defects definition

**Figure 2:**
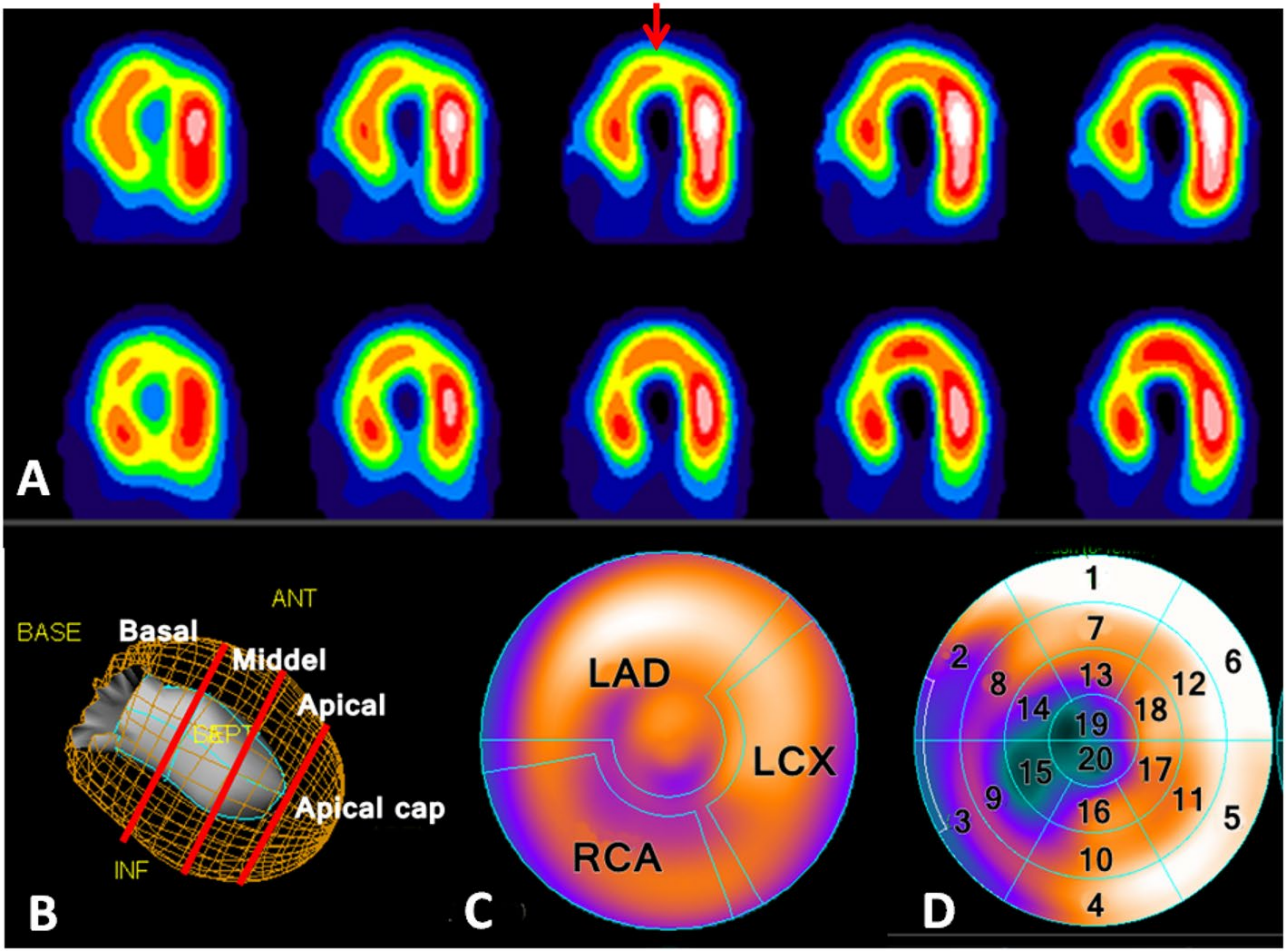
Demonstrations of myocardial perfusion analytic display of the study. (**A**) MPI SPECT multislice display of conventional qualitative interpretation, under post-stress (upper panel) and redistribution (lower panel) in the horizontal axis, respectively. The arrow shows the stress-induced perfusion defect with revere at apex. (**B**) Four rings display. LV myocardium was divided into four rings, namely basal, middle, apical, and apical cap. (**C**) Three territories display based on major coronary arteries supply. (**D**) Twenty segments display according to AHA’s 20-segment model. *MPI* myocardial perfusion imaging. *SPECT* single photon emission computed tomography. *LV* left ventricle. *AHA* American heart association.

We reconstructed the SPECT images by using filtered back projection and multi-slice display, which is shown in Fig. 2. Moreover, more precise segmental display including four rings, three territories and 20 segments were applied. Quantitative Perfusion SPECT and Gated SPECT software (Cedars-Sinai Medical Center, Los Angeles, CA) were used to quantify cardiac perfusion indexes, including summed stress score (SSS), summed rest score (SRS), and summed difference score (SDS)^19^.

We analyzed the perfusion results according to the AHA’s 20-segment model, and each patient’s myocardial perfusion was scored on a 5-point scale: (0) *normal myocardial perfusion*, (1) *mild myocardial perfusion defect*, (2) *moderate myocardial perfusion defect*, (3) *severe myocardial perfusion defect*, and (4) *no myocardial perfusion* (Table 2). Total scores of < 4, 4–8, 9–13, and > 13 were considered to have no, mild, moderate, or severe cardiac dysfunction, respectively. Each patient’s SSS, SRS, and SDS (the sum of the segment scores post-stress and redistribution, respectively) denoted changes in the patient’s overall cardiac blood flow. Patients whose SSS, SRS, or SDS before and after radiotherapy differed by ≥ 2 points and whose score for an individual segment of the LV increased by 2 or more points were determined to have new perfusion defects (NPD)^20^.

**Table 2:** Quantitation of myocardial perfusion defects definition.

5-point scale		Summed score for LV segments	
0	Normal myocardial perfusion	< 4	No cardiac dysfunction
1	Mild myocardial perfusion defect	4–8	Mild cardiac dysfunction
2	Moderate myocardial perfusion defect	9–13	Moderate cardiac dysfunction
3	Severe myocardial perfusion defect	> 13	Severe cardiac dysfunction
4	No perfusion		

SSS, SRS, or SDS before and after radiotherapy.

(1) Differed by ≥ 2 points,

(2) And score increased by 2 or more points for an individual segment, were determined to be NPD group.

*SSS* summed stress scores, *SRS* summed rest scores, *SDS* summed difference scores, *LV* left ventricle.

### Computed tomography simulation and radiation treatment planning

Each patient was confined in a torso immobilization vacuum pad (CIVCO Medical Instruments, Kalona, IA, USA) to simulate a typical position during breast cancer treatment. Scanning was conducted using a CT simulator (Discovery CT590 RT, GE Medical Systems, Amersham, UK) under the following conditions: a tube voltage of 120 kV, tube current of 300 mA, slice thickness of 2.5 mm, and pitch of 1.

**Figure 3:**
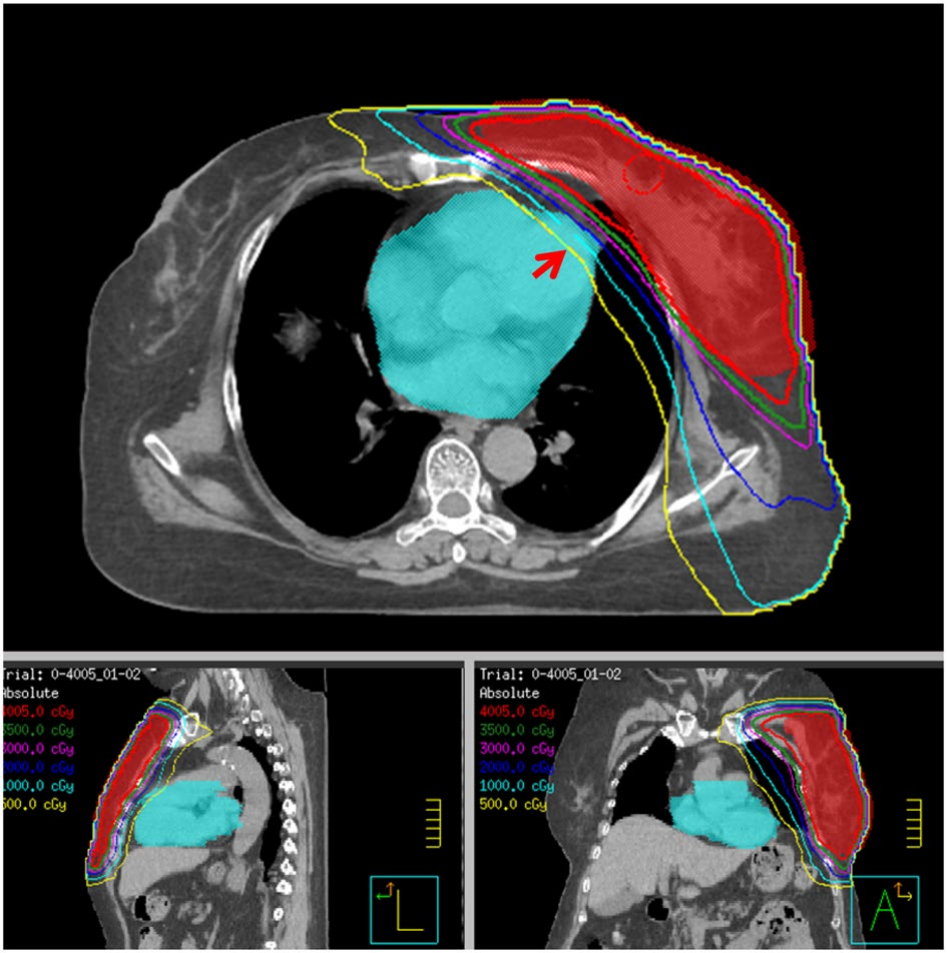
Schematic diagram of isodose curve in radiation therapy plan of a left breast cancer patient. The PTV and heart are depicted in red and blue colors, respectively. The isodose curve shows relative low but inevitable heart radiation involvement (arrow). *PTV* planning target volume.

The CT simulation images were input into the Pinnacle treatment planning system (version 9.8, Philips Medical Systems North America, Andover, MA, USA) to develop the radiation treatment planning (RTP). The planning target volume was the left breast, and the organs at risk included the right breast, LV, and lungs. The radiotherapy technologies applied were volumetric modulated arc therapy and intensity-modulated radiotherapy in Fig. 3. The CT simulation images and RTP were retrospectively collected.

### Radiation therapy techniques

During the RT treatment, the patients were positioned in a supine posture, ensuring free and unrestricted respiration. The comprehensive clinical tumor volume (CTV) encompassed the entirety of the breast or chest wall, along with the supraclavicular and infraclavicular regions, inclusive of any vulnerable segment within the axillary bed, as well as the possibility of incorporating the optional internal mammary nodes for regional nodal irradiation. Expanding upon the CTV, the planning target volume (PTV) was meticulously defined, accommodating a 5–8 mm margin to account for potential setup errors. The RT prescription comprised a conventional dosage of 45–50.4 Gy, divided into 25–28 fractions, or alternatively, a hypofractionated dose of 40–42.5 Gy, distributed over 15–16 fractions, while daily fractions were directed towards the breast or chest wall and regional nodal irradiation. Additionally, an optional boost dose of 10–16 Gy could be administered to the tumor bed or surgical scar.

With the objective of achieving enhanced homogeneity and conformity in target coverage, as well as minimizing exposure to neighboring normal organs, the planning encompassed two techniques: volumetric-modulated arc therapy and helical tomotherapy. These techniques were carefully employed to ensure the preservation of the heart and other organs at risk by diminishing their potential radiation exposure. In order to evaluate the adherence to dose constraints for the target volumes and other organs at risk, a dose-volume histogram was utilized. The criteria established were as follows: the CTV should receive a minimum of 100% of the prescribed dose, while the PTV should receive 95–100% of the prescribed dose for at least 95% of its volume. Additionally, it was imperative that the maximum dose administered to the PTV region remain below 110% of the prescribed dose. Constraints concerning the heart entailed a mean heart dose below 26 Gy, as well as a V20 value below 10%.

### The subvolume doses of the left ventricle acquisition

**Figure 4:**
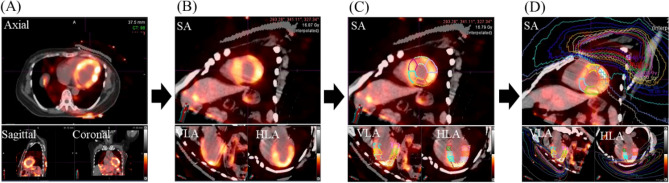
Workflow of generating the subvolume doses of LV: (**A**) To fuse SPECT MPI image with CT simulation image, with the LV as the reference for registration; (**B**) to rotate the images to SA view, VLA view, and HLA view, to fit the conventional MPI axes ; (**C**) To divided the LV into 20 segments in accordance with AHA’s 20-segment model; and (**D**) To input the dose distribution of radiotherapy plan to obtain the subvolume doses of the 20 segments separately. *LV* left ventricle. *SA* short axis. *VLA* vertical long axis. *HLA* horizontal long axis.

The CT simulation images and SPECT MPI images were fused and registered in the treatment planning system of radiation therapy, and the fused images were rotated to obtain a short axis (SA), vertical long axis (VLA), and horizontal long axis (HLA) view. We divided the myocardium of LV into four rings (namely the basal, middle, apical, and apical cap rings) and into 20 segments afterward (Fig. 4). The subvolume doses of the LV were acquired from the fused images.

### Statistical analyses

IBM SPSS version 20.0 was used for statistical analyses. The MHD, MLVD, the doses to four rings, the doses to three territories and doses of the 20 segments between the NPD group and non new perfusion defect (non-NPD) group were compared through a Mann–Whitney test. A *p* value of < 0.05 indicated statistical significance^21^.

### Ethics approval

The institutional review board of Far Eastern Memorial Hospital (No. 101085-F) approved this retrospective study and waived the need for written informed consent.

## Results

### Analysis of demographic data in NPD group and non-NPD group

The 61 patients were divided into two groups: the NPD group (n = 28) and the non-NPD group (n = 33) according to MPI quantitation results, as shown in Table 1. All the patients had normal left ventricular ejection fractions (LVEFs > 55%) before and after treatment; none of the patients exhibited significant LVEF decline. The median prescribed dose of radiotherapy for the patients in the NPD and non-NPD groups was the same, with 55.00 Gy (range, 50.00–61.00 Gy) and 55.00 Gy (range, 40.05–60.00 Gy), respectively. No significant intergroup differences in CAD risk indices were identified.

### The MHD, MLVD, the doses to four rings, the doses to three territories in the NPD and non-NPD group

As indicated in Table 3, the average MHD and mean LV dose (MLVD) were 3.14 and 4.84 Gy in the NPD group, respectively, and in the non-NPD group were 3.08 and 4.71 Gy, respectively. The average MHD (*p* = 0.863) and MLVD (*p* = 0.812) did not differ significantly between the groups. In the four rings analysis, the apical cap received the highest dose. Among the three territories (the LAD, right coronary artery [RCA], and left circumflex artery [LCX]), the LAD received the highest dose of radiation. The MHD, MLVD, the doses to the four rings, and the doses to the three territories in the NPD group were higher than those in the non-NPD group, although none of the differences reached statistical significance.

**Table 3:** The MHD, MLVD, the doses to four rings, the doses to three territories in the NPD and non-NPD groups.

Structure	Mean ± SD (Gy)			*p* value
	All patients (n = 61)	NPD (n = 28)	non-NPD (n = 33)	
Heart	3.11 ± 1.47	3.14 ± 1.64	3.08 ± 1.35	0.863
Left ventricle	4.77 ± 1.97	4.84 ± 2.21	4.71 ± 1.81	0.812
Basal	2.47 ± 1.13	2.76 ± 1.41	2.21 ± 0.77	0.065
Middle	3.82 ± 1.92	4.10 ± 2.37	3.59 ± 1.45	0.313
Apical	7.27 ± 3.91	7.72 ± 4.63	6.87 ± 3.25	0.405
Apical cap	15.32 ± 7.67	15.98 ± 8.25	14.74 ± 7.33	0.538
Territory				
LAD	9.08 ± 4.76	9.68 ± 5.93	8.57 ± 3.56	0.375
RCA	2.24 ± 1.03	2.34 ± 1.04	2.15 ± 1.05	0.490
LCX	4.79 ± 2.28	5.00 ± 2.83	4.61 ± 1.72	0.512

*NPD* new perfusion defect, *non-NPD* non new perfusion defect, *LAD* left anterior descending artery, *LCX* left circumflex artery, *RCA* right coronary artery.

### Comparison of subvolume doses of the left ventricle between the NDP and non-NDP group

**Figure 5:**
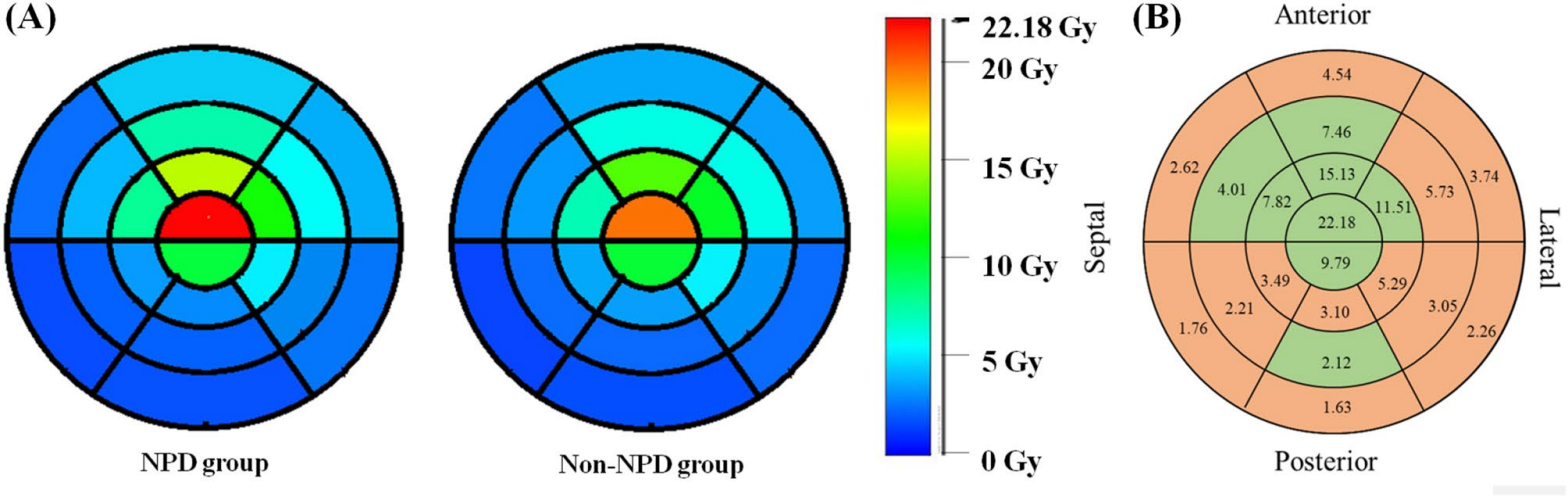
The subvolume dose distribution of 20 segments in the bull's eye plot display. (**A**) Using gradient rainbow color scale to show the mean subvolume dose distribution in NPD group (left), and non-NPD group (right), respectively. (**B**) Ischemia segments distribution of the NPD group. The numbers represents the average subvolume dose (Gy) of each segment. The orange areas display the presence of the new perfusion segments, while the green areas show the segments without perfusion decline. *NPD* new perfusion defect. *Non-NPD* non new perfusion defect.

The mean subvolume doses of the 20 segments in NPD group were all higher than the non-NPD group generally (Table 4). A significant difference in subvolume dose in segment 3 (*p* = 0.020) was observed. As to the segment 19, which received the highest subvolume dose (22.18 ± 10.38 Gy), the dose difference does not reach significance in NPD and non-NPD group. Figure 5A demonstrates the subvolume dose with gradient color scale, the two groups show relatively similar decreasing fashions of subvolume dose, expect for remarked higher dose at segment 19 and 13. The radiation doses and NPD areas in the NPD group were presented in the bull's eye plot as shown in Fig. 5B. Most of the areas with defects were located in the basal ring, and the average dose to the basal ring was 2.75 Gy. Among the four rings, the basal ring also received the lowest dose. Concerning the condition of overall myocardial ischemia existence by dividing the myocardial ischemia status with NPD and non-NPD group, the result may suggest that even the non-significant difference of subvolume dose may contribute to perfusion decline.

**Table 4:** Doses to 20 segments of the left ventricle in NPD and non-NPD group.

Segment	Mean ± SD (Gy)			*p* value
	All patients (n = 61)	NPD (n = 28)	non-NPD (n = 33)	
Basal				
1	3.87 ± 2.69	4.54 ± 3.53	3.29 ± 1.54	0.075
2	2.37 ± 1.23	2.62 ± 1.46	2.16 ± 0.99	0.153
3	1.56 ± 0.61	1.76 ± 0.78	1.39 ± 0.35	0.020*
4	1.52 ± 0.60	1.63 ± 0.59	1.42 ± 0.61	0.183
5	2.09 ± 0.90	2.26 ± 1.10	1.94 ± 0.69	0.186
6	3.38 ± 1.90	3.74 ± 2.52	3.08 ± 1.12	0.184
Middle				
7	6.69 ± 4.67	7.46 ± 5.92	6.02 ± 3.28	0.243
8	3.58 ± 1.99	4.01 ± 2.51	3.20 ± 1.36	0.119
9	2.06 ± 0.95	2.21 ± 0.88	1.94 ± 1.01	0.267
10	2.05 ± 1.14	2.12 ± 1.07	1.99 ± 1.23	0.677
11	2.99 ± 1.42	3.05 ± 1.70	2.94 ± 1.18	0.788
12	5.57 ± 3.24	5.73 ± 3.83	5.42 ± 2.74	0.721
Apical				
13	13.89 ± 8.32	15.13 ± 9.90	12.81 ± 6.80	0.289
14	7.32 ± 5.18	7.82 ± 6.06	6.87 ± 4.42	0.487
15	3.38 ± 2.14	3.49 ± 2.04	3.29 ± 2.28	0.732
16	3.08 ± 1.89	3.10 ± 1.85	3.07 ± 1.98	0.946
17	5.17 ± 3.08	5.29 ± 3.48	5.07 ± 2.78	0.785
18	10.75 ± 5.89	11.51 ± 7.28	10.09 ± 4.47	0.359
Apical cap				
19	20.85 ± 9.95	22.18 ± 10.38	19.69 ± 9.74	0.341
20	9.79 ± 6.12	9.79 ± 6.57	9.79 ± 5.91	0.998

*NPD* new perfusion defect, *non-NPD* non new perfusion defect.

Values were presented as mean ± SD.

*Represents significant difference (*p* < 0.05).

## Discussion

In the present study, we successfully integrated the LV subvolume doses with the SPECT MPI cardiac perfusion and function indices of the corresponding segments in the bull's eye plot. Few studies have investigated the relationship between myocardial perfusion defects and subvolume radiotherapy doses in patients with breast cancer after therapy. The doses to each of the 20 segments in the NPD group were higher than those in the non-NPD group.

SPECT MPI was well validated to investigate early changes in the patients’ cardiac perfusion function at the molecular level. Among the 61 patients, 10 had a high myocardial perfusion defect score (> 4 points) before radiotherapy. Before radiotherapy, 37 (61%), 8 (13%), and 39 (64%) patients had undergone chemotherapy, target therapy, and hormone therapy, respectively. Moslehi^22^ reported that breast cancer treatments, such as radiotherapy, chemotherapy, and targeted therapy, can cause cardiovascular damage and changes in myocardial perfusion. It is known that radiotherapy was not the only factor that could induce changes in cardiac function and that other breast cancer treatments also affect the heart.

MHD has identified a linear relationship of developing cardiovascular disease after treatment, which increases by 7.4% with each additional 1 Gy of MHD^1^. Moreover, no threshold exists for cardiac dysfunction or cardiovascular disease incidence. That is, cardiac dysfunction and cardiovascular disease can still occur as a result of radiation in low doses. We observed that NPDs occurred in segments 1, 2, 3, 4, 5, 6, 8, 9, 11, 12, 15, 16, and 17, all of which received low radiation doses. Segments that receive low doses of radiation may also exhibit defects, which is consistent with previously reported results.

As indicated in the bull’s eye plot, the patients’ apical cap rings received high doses of radiation (Fig. 5B) but did not exhibit NPDs, revealing that the apical cap may have relatively high radiation resistance. Another possible explanation for this trend is that the location of the apical cap changes during the cardiac cycle, causing the apical cap to receive less irradiation than the PTV.

Among the 20 segments of the LV, segments 19, 13, 18, 20, and 14 received the highest doses of radiation. Tang et al.^17^ divided the LV according to the AHA’s 17-segment model and determined that segments 17, 13, 16, 14, and 7 received the largest doses of radiation. A comparison of Tang’s findings and those of the present study suggests that segments receiving high radiation doses were in close proximity, with the highest doses received by the apical ring and apical cap ring in both studies. In the present study, the SPECT MPI cardiac function indices before and after treatment were combined to more accurately assess the effects of different doses on each segment in the 20-segment model.

Wennstig et al.^23^ recruited 182 patients who received radiotherapy for Hodgkin lymphoma and discovered a correlation between radiation dose to the coronary arteries and coronary artery stenosis, the incidence of which increased by 4.9% with each additional 1 Gy of radiation to the coronary arteries. Therefore, we assessed the doses received by the LAD, RCA, and LCX in the NPD and non-NPD groups, which were 9.68, 2.34, and 5.00 Gy, respectively, in the NPD group and 8.57, 2.15, and 4.61 Gy, respectively, in the non-NPD group. The NPD group received higher doses to each of the three territories than did the non-NPD group. Although most of the segments of the LAD received high radiation doses, no NPDs were found in six of the 11 segments in the NPD group.

Radiation-induced heart diseases are generally chronic. Marks et al.^24^ analyzed the cardiac function of 114 patients in their study, in which SPECT examinations were performed 6, 12, 18, and 24 months after radiotherapy. The proportions of patients with new cardiac dysfunction at each time point were 27%, 29%, 38%, and 42%, respectively. Darby et al.^1^ conducted a 20 years follow-up study and reported that patients can develop cardiovascular diseases 0–4 years, 5–9 years, 10–19 years, and ≥ 20 years after radiotherapy. The incidence of cardiovascular disease occurring within 0–4 years after radiotherapy increases by 16.4% with each additional 1 Gy of MHD. Although modern RT techniques have been known to optimize target coverage and dose conformity, the cardiac mortality rate relates to radiation dose^25^. The acute effects include inflammatory cell infiltration and endothelial damage, and the chronic heart diseases may develop 12 years after treatment, such as radiation-induced pericardial disease^8^. This study collected SPECT MPI images of patients with left-breast cancer taken 1 year after radiotherapy in an attempt to detect myocardial defects at an early stage. Using molecular level myocardial perfusion scan to detect the early cardiac dysfunction may be helpful to detect the subtle injury degree, or predict future possible chronic injury. In addition, patients with breast cancer are subject to a significantly higher coronary artery calcium burden after adjuvant radiotherapy^26^.

Our results indicate that current treatment strategies may provide fair cardiac safety in general perfusion and function during the follow-up period, but subtle radiation dose-related injuries does exist. Therefore, cardiovascular physicians and patients with breast cancer should be aware of the long-term cardiac risks after regional or systemic anticancer therapy.

This study has several limitations. First, the simulation CT images were not acquired through electrocardiogram-gated acquisition. Cardiac motion during the cardiac cycles may affect cardiac contouring or image fusion. In future studies, researchers can obtain dynamic cardiac images through electrocardiogram-gated acquisition and fuse them with images from the eight phases of the cardiac cycle to minimize errors in image fusion. Electrocardiogram-gated planning may reduce the radiation dose to nontarget tissues. Second, some of the patients had perfusion defects before radiotherapy but exhibited perfusion recovery after radiotherapy. Some other medical conditions may contribute to changes in myocardial perfusion, including postoperative soft-tissue inflammation resulting in photon attenuation or transient postchemotherapy cardiac toxicity.

## Conclusion

In this study, the subvolume doses to the 20 segments of the LV in the NPD group were higher than those in the non-NPD group significantly at segment 3 basal inferoseptal region, and higher in other segments in general. According to the bull’s eye plot of radiation dose and NPD area, new cardiac dysfunction might occur even in patients treated with low doses of radiation. The bull’s eye plot presented herein may serve as a reference for clinical physicians to precisely assess subtle cardiac function perfusion decline caused by irradiation in each segment.

## Data Availability

The datasets used and/or analyzed during the current study are available from the corresponding author on reasonable request.

## Abbreviations

SPECT: Single photon emission computed tomography
MPI: Myocardial perfusion imaging
MHD: Mean heart dose
LAD: Left anterior descending artery
RCA: Right coronary artery
LCX: Left circumflex artery
LV: Left ventricle
AHA: American heart association
CAD: Coronary artery disease
SSS: Summed stress score
SRS: Summed rest score
SDS: Summed difference score
NPD: New perfusion defect
non-NPD: Non new perfusion defect found
RTP: Radiation treatment planning
PTV: Planning target volume
SA: Short axis
VLA: Vertical long axis
HLA: Horizontal long axis
MLVD: Mean left ventricle dose
